# Supplementary material for: Responses of Physiology, Photosynthesis, and Related Genes to Saline Stress in Cornus hongkongensis subsp. tonkinensis (W. P. Fang) Q. Y. Xiang
Source: Plants (Basel). 2022 Mar 30;11(7):940. doi: 10.3390/plants11070940 (PMC9002922; doi:10.3390/plants11070940)
Supplement: Supplementary file 1 [file plants-11-00940-s001.zip › plants-1633829-supplementary.pdf]

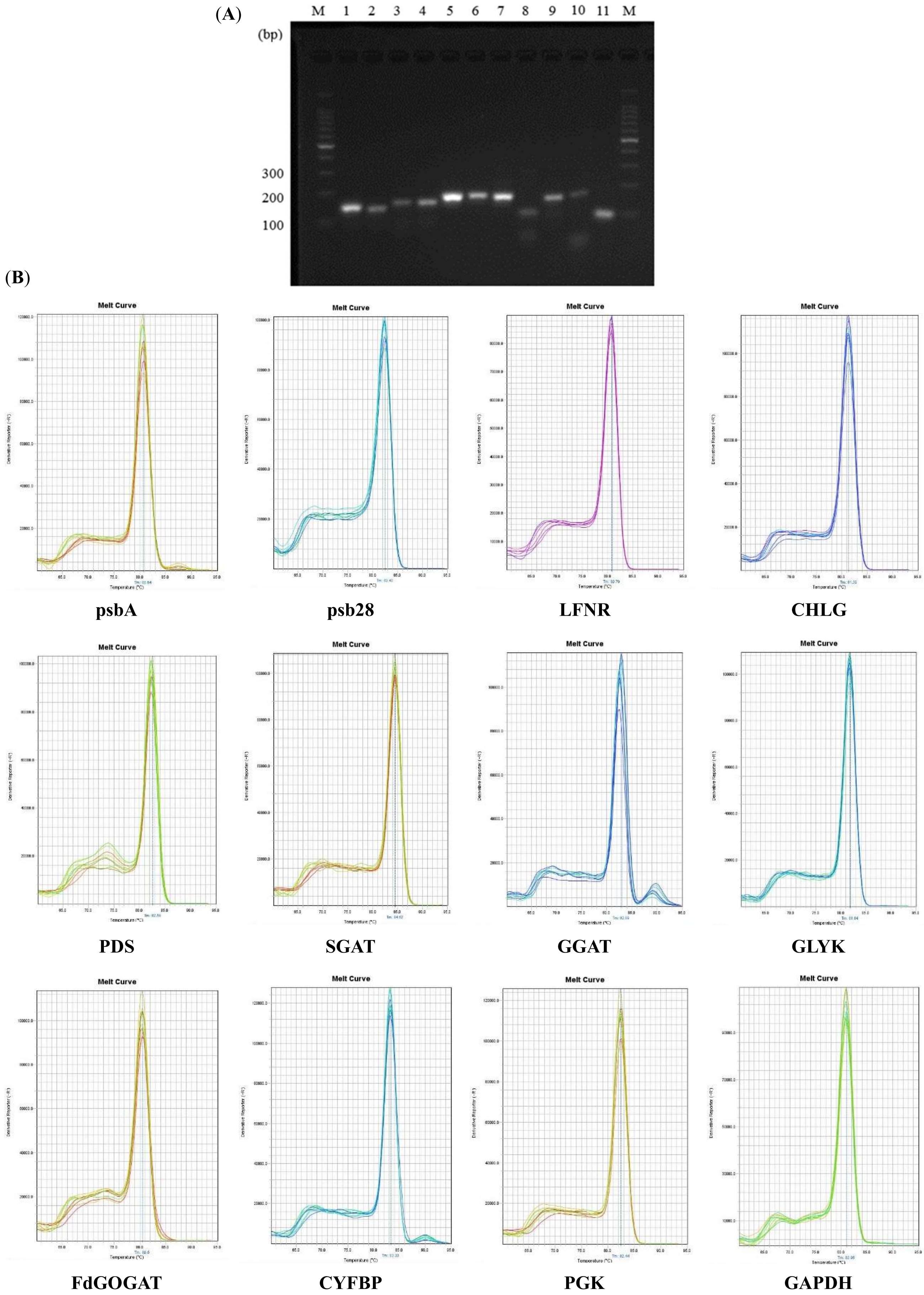

A: 1. psbA, 2. psb28, 3. LFNR, 4. SGAT, 5. GGAT, 6. GLKY, 7. CYFBP, 8. FdGOGAT, 9. PGK, 10. PDS, 11. CHLG, M: Marker 100bp

Figure S1. Products of photosynthesis and photorespiration genes (A) and melt curve amplified by qRT-PCR (B).

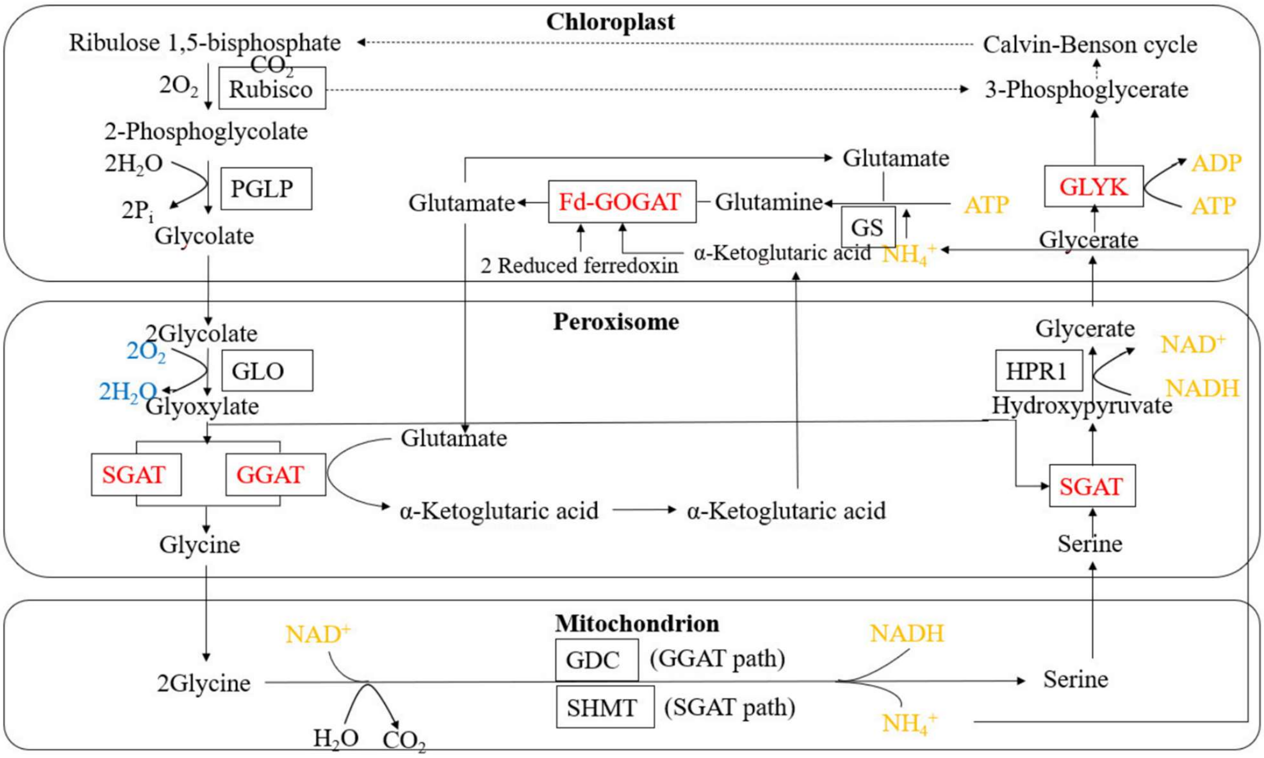

Note: The involved key enzymes in the photorespiration pathway in our study were marked in red. The dotted line represents the carboxylation pathway catalyzed by Rubisco, and the solid line represents the oxygenation pathway catalyzed by Rubisco. PGLP, 2-phosphoglycolate phosphatase; GLO, Glycolate oxidase; SGAT, Serine: glyoxalate aminotransferase; GGAT, Glutamate: glyoxalate aminotransferase; GDC, Glycine decarboxylase complex; SHMT, Serine hydroxymethyltransferase; HPR1, Hydroxy pyruvate reductase 1; GLYK, Glycerate kinase; Fd-GOGAT, Ferredoxin-dependent glutamate synthase.

Figure S2. Schematic diagram of photorespiration metabolic pathway [13].

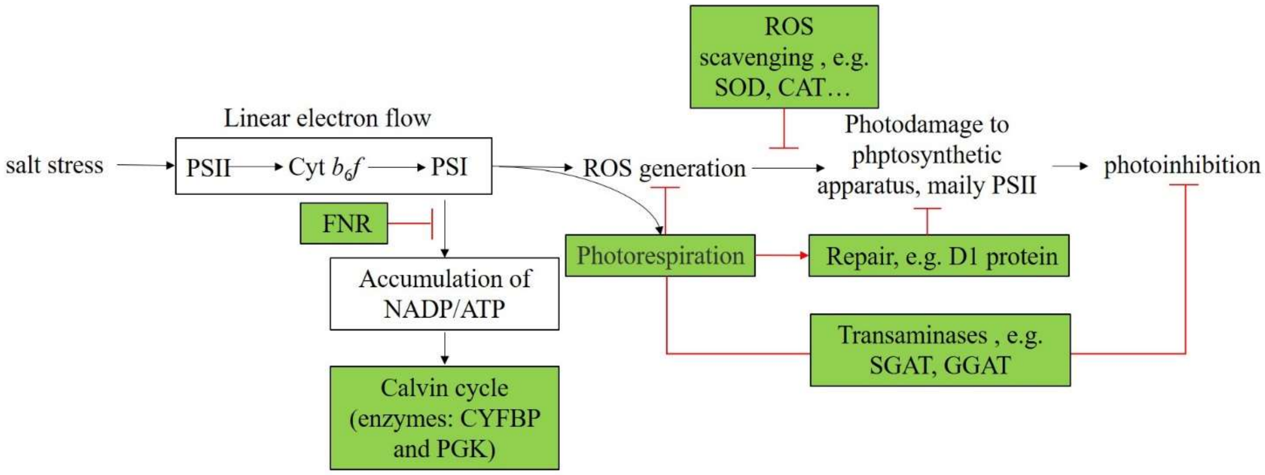

Note: The green boxes indicate protective mechanisms. The black arrow indicates a causal link while the red line indicates a positive effect. Reproduced with permission of Ref. Enhancing photosynthesis in plants: The light reactions., Copyright of Cardona, T.; Shao, S.X.; Nixon, P.J, Essays Biochem, 2018.

Figure S3. Partial relationship between photosynthetic electron flow, photodamage, and photoprotection (Cardona et al., 2008).

Table S1. List of primers for qRT-PCR of candidate genes.

Genes names	The sequence of primer (5'-3')	Expected product size/bp	Protein names
psbA-F	TCGCCTTCATTGCTGCTCCTCC	150	Photosystem II protein D1
psbA-R	CCGCTTCCCAGATCGGGTAAAA		
psb28-F	ACTCGATTTCTTTCAGGCATTG	137	Photosystem II reaction center Psb28 protein
psb28-R	ACCGTAAGTTTAGGCATTTGGA		
LFNR-F	AGAACAAACTAATGACAAAGGCG	154	Ferredoxin--NADP reductase, leaf-type isozyme, chloroplastic
LFNR-R	CCCCATTATATCGTCAATCCCC		
SGAT-F	AAAGGTGTTCAGGATAGGGC	154	Serine--glyoxylate aminotransferase
SGAT-R	AATGGTGTTCTGTAGGTACGC		
GGAT-F	CCTCGCCAGGTGATTGCTCTAT	180	Glutamate--glyoxylate aminotransferase
GGAT-R	CTCGGCCACTTCCTCCCTTATT		
GLYK-F	TTCCCACTGAAGTCATCAAAGC	178	D-glycerate 3-kinase
GLYK-R	CCATCTGCCCTCATAGCAATCT		
CYFBP-F	AAGGCAGGTCTTGCTAAACTCA	174	Fructose-1,6-bisphosphatase, cytosolic
CYFBP -R	CGGCTCCACAAATGTTGCTTCT		
FdGOGAT-F	GACTATGTGGGAAAGGGTATGG	112	Ferredoxin-dependent glutamate synthase
FdGOGAT-R	CCCCATATAAGCAGGTATTCCC		
PGK-F	TTGTGGCTGCACTTCCTGATGG	165	Phosphoglycerate kinase, chloroplastic-like isoform X2
PGK-R	AGTTGAGGCGTGGGCTCTATGT		
PDS-F	TGAAATGCTTACTTGGCCTGAG	171	Phytoene desaturase
PDS-R	TTTGACATGGCAATGAACACCT		
CHLG-F	AAAAGTGTTGAAGGGGATAGAG	103	Chlorophyll synthase
CHLG-R	GAGTAACGTCAATAGCACCAAC		

Table S2. Pearson correlation coefficients for photosynthetic characteristics and the growth leaves in *C. hongkongensis* subsp. *tonkinensis*.

	Pn	gs	Ci	Tr	WUE	Fv/Fm	Fv/Fo	Chl a	Chl b	Car	T Chl	Chla/b	SDLs	MDA	REC	LRWC	SOD	CAT	PRO	SS	SP
**Pn**	1	0.865**	0.006	0.807**	0.596**	0.598**	0.678**	0.320*	0.071	0.251	0.249	0.496**	-0.899**	-0.580**	-0.616**	0.585**	-0.148	-0.413**	-0.541**	-0.700**	-0.593**
**gs**	0.865**	1	0.401**	0.877**	0.296	0.530**	0.599**	0.241	0.067	0.205	0.191	0.293	-0.774**	-0.601**	-0.563**	0.481**	-0.242	-0.162	-0.475**	-0.612**	-0.522**
**Ci**	0.006	0.401**	1	0.208	-0.326*	-0.002	-0.018	-0.094	-0.065	-0.064	-0.086	-0.079	0.053	-0.278	-0.154	-0.087	-0.168	0.142	0.022	0.029	0.015
**Tr**	0.807**	0.877**	0.208	1	0.075	0.525**	0.608**	0.496**	0.332*	0.467**	0.453**	0.181	-0.766**	-0.466**	-0.455**	0.385*	-0.255	-0.036	-0.476**	-0.581**	-0.475**
**WUE**	0.596**	0.296	-0.326*	0.075	1	0.423**	0.432**	-0.129	-0.376*	-0.175	-0.207	0.694**	-0.553**	-0.466**	-0.533**	0.571**	0.004	-0.686**	-0.402*	-0.441**	-0.494**
**Fv/Fm**	0.598**	0.530**	-0.002	0.525**	0.423**	1	0.966**	0.093	-0.164	0.102	0.015	0.477**	-0.808**	-.0718**	-0.828**	0.870**	-0.416**	-0.182	-0.891**	-0.886**	-0.763**
**Fv/Fo**	0.678**	0.599**	-0.018	0.608**	0.432**	0.966**	1	0.131	-0.117	0.109	0.056	0.458**	-0.879**	-0.726**	-0.852**	0.865**	-0.407**	-0.21	-0.870**	-0.860**	-0.798**
**Chla**	0.320*	0.241	-0.094	0.496**	-0.129	0.093	0.131	1	0.924**	0.939**	0.993**	-0.098	-0.277	-0.131	0.014	-0.051	-0.13	0.175	-0.013	-0.167	-0.103
**Chlb**	0.071	0.067	-0.065	0.332*	-0.376*	-0.164	-0.117	0.924**	1	0.861**	0.963**	-0.456**	0	0.056	0.234	-0.289	-0.068	0.403**	0.227	0.055	0.137
**Car**	0.251	0.205	-0.064	0.467**	-0.175	0.102	0.109	0.939**	0.861**	1	0.930**	-0.076	-0.237	-0.157	0.009	-0.057	-0.228	0.257	-0.017	-0.134	-0.037
**T Chla**	0.249	0.191	-0.086	0.453**	-0.207	0.015	0.056	0.993**	0.963**	0.930**	1	-0.21	-0.196	-0.075	0.082	-0.125	-0.113	0.248	0.061	-0.101	-0.03
**Ca/Cb**	0.496**	0.293	-0.079	0.181	0.694**	0.477**	0.458**	-0.098	-0.456**	-0.076	-0.21	1	-0.543**	-0.345**	-0.455**	0.492**	-0.067	-0.708**	-0.455**	-0.363*	-0.498**
**SDL**	-0.899	-0.774	0.053	-0.766	-0.553	-0.808	-0.879	-0.277	0	-0.237	-0.196	-0.543	1	0.652**	0.757**	-0.780	0.237	0.327*	0.750**	0.812**	0.780**

	**	**		**	**	**	**					**				**					
**MDA**	-0.580**	-0.601**	-0.278	-0.466**	-0.466**	-0.718**	-0.726**	-0.131	0.056	-0.157	-0.075	-0.345*	0.652**	1	0.858**	-0.716**	0.436**	0.171	0.666**	0.663**	0.537**
**REC**	-0.616**	-0.563**	-0.154	-0.455**	-0.533**	-0.828**	-0.852**	0.014	0.234	0.009	0.082	-0.455**	0.757**	0.858**	1	-0.863**	0.436**	0.259	0.746**	0.713**	0.650**
**LRWC**	0.585**	0.481**	-0.087	0.385*	0.571**	0.870**	0.865**	-0.051	-0.289	-0.057	-0.125	0.492**	-0.780**	-0.716**	-0.863**	1	-0.232	-0.26	-0.854**	-0.817**	-0.747**
**SOD**	-0.148	-0.242	-0.168	-0.255	0.004	-0.416**	-0.407**	-0.13	-0.068	-0.228	-0.113	-0.067	0.237	0.436**	0.436**	-0.232	1	-0.057	0.192	0.301	0.184
**CAT**	-0.413**	-0.162	0.142	-0.036	-0.686**	-0.182	-0.21	0.175	0.403**	0.257	0.248	-0.708**	0.327*	0.171	0.259	-0.26	-0.057	1	0.174	0.181	0.294
**PRO**	-0.541**	-0.475**	0.022	-0.476**	-0.402*	-0.891**	-0.870**	-0.013	0.227	-0.017	0.061	-0.455**	0.750**	0.666**	0.746**	-0.854**	0.192	0.174	1	0.791**	0.711**
**SS**	-0.700**	-0.612**	0.029	-0.581**	-0.441**	-0.886**	-0.860**	-0.167	0.055	-0.134	-0.101	-0.363*	0.812**	0.663**	0.713**	-0.817**	0.301	0.181	0.791**	1	0.717**
**SP**	-0.593**	-0.522**	0.015	-0.475**	-0.494**	-0.763**	-0.798**	-0.103	0.137	-0.037	-0.03	-0.498**	0.780**	0.537**	0.650**	-0.747**	0.184	0.294	0.711**	0.717**	1

*and** indicate that correlation is significant at the 0.05 and 0.01 level, respectively.

Table S3. Pearson correlation coefficients for photosynthetic parameters and related gene expression in *C. hongkongensis* subsp. *tonkinensis*.

	P_n	gs	C_i	T_r	WUE	F_v/F_m	F_v/F_o	Chla	Chlb	Car	TChl	Chla/b	PsbA	Psb28	LFNR	CHLG	PDS	SGAT	GGAT	GLYK	FdGOGAT	CYFBP	PGK
**P_n**	1	0.810**	0.121	0.629**	0.698**	0.561*	0.519*	0.288	-0.083	0.142	0.179	0.461	0.152	0.027	0.108	-0.581*	0.211	0.099	0.090	-0.082	0.356	-0.039	0.036
**gs**	0.810**	1	0.582*	0.890**	0.205	0.380	0.365	0.315	0.170	0.212	0.283	0.101	0.023	0.269	0.214	-0.456	0.494*	-0.199	0.005	-0.004	0.298	0.032	0.055
**C_i**	0.121	0.582*	1	0.460	-0.343	-0.336	-0.318	-0.169	-0.073	-0.155	-0.146	-0.128	-0.366	0.097	-0.188	-0.067	0.321	-0.275	-0.321	-0.092	0.104	-0.031	-0.262
**T_r**	0.629**	0.890**	0.460	1	-0.099	0.468	0.464	0.425	0.444	0.373	0.454	-0.173	0.042	0.386	0.436	-0.366	0.710**	-0.461	0.124	0.103	0.056	0.069	0.171
**WUE**	0.698**	0.205	-0.343	-0.099	1	0.348	0.289	0.076	-0.443	-0.051	-0.094	0.768**	0.195	-0.325	-0.187	-0.401	-0.406	0.578*	0.047	-0.175	0.418	-0.063	-0.076
**F_v/F_m**	0.561*	0.380	-0.336	0.468	0.348	1	0.992**	0.443	0.285	0.257	0.414	0.093	0.602**	0.270	0.491*	-0.301	0.306	0.095	0.466	0.247	-0.024	0.190	0.493*
**F_v/F_o**	0.519*	0.365	-0.318	0.464	0.289	0.992**	1	0.418	0.298	0.234	0.401	0.036	0.592**	0.312	0.503*	-0.330	0.353	0.044	0.483*	0.266	-0.100	0.178	0.513*
**Chla**	0.288	0.315	-0.169	0.425	0.076	0.443	0.418	1	0.769**	0.773**	0.977**	0.074	0.259	0.043	0.277	-0.044	0.013	-0.297	-0.110	-0.301	0.375	-0.182	0.022
**Chlb**	-0.083	0.170	-0.073	0.444	-0.443	0.285	0.298	0.769**	1	0.587*	0.888**	-0.576*	0.215	0.306	0.494*	0.246	0.278	-0.485*	0.034	0.007	0.122	0.040	0.239
**Car**	0.142	0.212	-0.155	0.373	-0.051	0.257	0.234	0.773**	0.587*	1	0.753**	0.108	0.207	0.183	0.305	-0.046	0.202	-0.404	-0.064	-0.170	0.204	-0.138	0.088
**TChl**	0.179	0.283	-0.146	0.454	-0.094	0.414	0.401	0.977**	0.888**	0.753**	1	-0.140	0.258	0.134	0.365	0.051	0.103	-0.376	-0.067	-0.214	0.310	-0.118	0.096
**Chla/b**	0.461	0.101	-0.128	-0.173	0.768**	0.093	0.036	0.074	-0.576*	0.108	-0.140	1	-0.001	-0.418	-0.406	-0.396	-0.426	0.380	-0.188	-0.384	0.298	-0.275	-0.332
**PsbA**	0.152	0.023	-0.366	0.042	0.195	0.602**	0.592**	0.259	0.215	0.207	0.258	-0.001	1	0.530*	0.580*	0.332	0.036	0.262	0.507*	0.405	0.188	0.323	0.730**
**Psb28**	0.027	0.269	0.097	0.386	-0.325	0.270	0.312	0.043	0.306	0.183	0.134	-0.418	0.530*	1	0.702**	0.133	0.632**	-0.259	0.552*	0.572*	-0.105	0.334	0.765**
**LFNR**	0.108	0.214	-0.18	0.436	-0.187	0.491*	0.503*	0.277	0.494*	0.305	0.365	-0.406	0.580*	0.702**	1	0.188	0.480*	-0.01	0.823**	0.758**	-0.172	0.622**	0.873**

			8															5					
**CHLG**	-0.581*	-0.456	-0.067	-0.366	-0.401	-0.301	-0.330	-0.044	0.246	-0.046	0.051	-0.396	0.332	0.133	0.188	1	-0.260	0.180	0.089	0.244	0.195	0.322	0.293
**PDS**	0.211	0.494*	0.321	0.710**	-0.406	0.306	0.353	0.013	0.278	0.202	0.103	-0.426	0.036	0.632**	0.480*	-0.260	1	-0.585*	0.264	0.443	-0.360	0.236	0.406
**SGAT**	0.099	-0.199	-0.275	-0.461	0.578*	0.095	0.044	-0.297	-0.485*	-0.404	-0.376	0.380	0.262	-0.259	-0.015	0.180	-0.585*	1	0.320	0.316	0.251	0.474*	0.220
**GGAT**	0.090	0.005	-0.321	0.124	0.047	0.466	0.483*	-0.110	0.034	-0.064	-0.067	-0.188	0.507*	0.552*	0.823**	0.089	0.264	0.320	1	0.807**	-0.384	0.605**	0.858**
**GLYK**	-0.082	-0.004	-0.092	0.103	-0.175	0.247	0.266	-0.301	0.007	-0.170	-0.214	-0.384	0.405	0.572*	0.758**	0.244	0.443	0.316	0.807**	1	-0.307	0.876**	0.863**
**FdGOGAT**	0.356	0.298	0.104	0.056	0.418	-0.024	-0.100	0.375	0.122	0.204	0.310	0.298	0.188	-0.105	-0.172	0.195	-0.360	0.251	-0.384	-0.307	1	0.056	-0.141
**CYFBP**	-0.039	0.032	-0.031	0.069	-0.063	0.190	0.178	-0.182	0.040	-0.138	-0.118	-0.275	0.323	0.334	0.622**	0.322	0.236	0.474*	0.605**	0.876**	0.056	1	0.706**
**PGK**	0.036	0.055	-0.262	0.171	-0.076	0.493*	0.513*	0.022	0.239	0.088	0.096	-0.332	0.730**	0.765**	0.873**	0.293	0.406	0.220	0.858**	0.863**	-0.141	0.706**	1

*and** indicate that correlation is significant at the 0.05 and 0.01 level, respectively.
